# Smallpox at Nottingham

**Published:** 1921-12

**Authors:** 


### SMALLPOX AT NOTTINGHAM.

**(From a Correspondent.)**

ON February 26 of this year a case of smallpox was diagnosed at Nottingham. The case was isolated, and the contacts, as far as they could be traced, were vaccinated and kept under observation. No infection apparently arose from this first case; and nothing more was heard of the disease in Nottingham until May 31, when a second case occurred. Since secondary cases of smallpox develop within a fortnight of the first case it is difficult to understand how this second case could have been infected by the patient who was ill three months earlier, unless we believe either that fresh infection was introduced into the city, or that the disease was kept going for three months by a series of missed cases, which seems unlikely. One month after the second case another person developed smallpox—on June 30—and a fourth case was reported on July 9. On July 12 Dr. W. V. Shaw, one of the medical officers on the staff at the Ministry of Health, was sent to Nottingham to investigate the outbreak and to give help to the local authority in controlling the spread of the disease.

At one time during the outbreak it seemed that smallpox was likely to become epidemic; but fortunately the disease did not spread rapidly. The type was very mild and no deaths occurred; indeed, except for a few of the older patients, no one was seriously ill. Owing to the number of missed cases which were not diagnosed until late, smallpox had every chance of becoming epidemic; and the fact that no epidemic occurred can only be attributed to the assumption that the disease is a very different one from what it was formerly, and that certainly it is not so infectious as it was in other days. This marked change of type may be due to the fact that for some thirty years or more the people of this country have been, on the whole, vaccinated; and that the smallpox virus passing through this relatively resistant population has been reduced in virulence, because the soil of those vaccinated people was unsuitable for it. Anthrax and other micro-organisms when grown upon culture media that are unsuitable to their rapid growth become non-virulent; and the same may have happened to the virus of smallpox.

During the week ending July 15 there were six cases of smallpox in Nottingham; in the following week, seven; and in the following week, ten. During the week ending August 6 there were five cases, and in the week ending August 13 there were eighteen cases. In the next week, four; and in the last week in August, six. During the same period the districts around Nottingham suffered as follows: Long Eaton Urban District had twelve cases, and four other outlying districts had one case each.

The condition of eighty-three smallpox patients regarding vaccination was as follows: Seventeen showed signs of vaccination, and the remainder (sixty-six) showed no evidence of vaccination The ages of the vaccinated persons who suffered from smallpox lay between twenty-two and sixty-eight years. Twenty-five males and twenty-eight females were attacked. Forty-one of the patients were under thirteen years of age, and forty were over that age.

Regarding the distribution in dwelling-houses it is very interesting to notice that only three cases occurred in the "slum" area of the city, and that the better-class houses seemed to suffer more heavily. The disease, however, was scattered over the city, and no one part was especially infected. Of the actual house-distribution of the disease, eleven houses had each two cases, three houses had three cases, one house four cases, and one house seven cases. Several of the smallpox patients worked on the railways or were associated with railway work of some description.

A census was taken at the schools to discover the number of non-vaccinated children, and it was found on inquiry that no less than 42 per cent. of the children were not vaccinated. Again, unless we are to believe that smallpox has changed its type and virulence, it is difficult to understand why a vast outbreak did not occur, especially among these unvaccinated children.

The disease was kept alive by mild, unrecognised, "missed" or undiagnosed cases; and the presence of an outbreak of undoubted chicken-pox, side by side with the smallpox, made the diagnosis of cases a matter sometimes of extreme difficulty. As an example of the danger of these missed or undiagnosed cases, the following can be considered typical: In the Clapton Road area of the Meadows district of Nottingham there lived three children, two of whom were called S—— and one T——. They suffered from unrecognised smallpox, and gave the infection to two unvaccinated children, H——, who in their turn infected an adult of fifty-two years.

Again, there was a girl, F——, who worked in a factory. She was attacked by smallpox in June, and was seen by a doctor. He diagnosed the case as chicken-pox, and informed the girl's employers as to his opinion. She infected a fellow-employee, M—— B——, and probably also a male machine-mender aged forty-seven. This man happened, in his spare time, to keep a tobacconist's shop in a busy street. He infected his daughter and perhaps some of his customers. These missed cases caused the disease to be prevalent.

In one or two instances the very dangerous practice was followed of keeping cases at home, although there was no doubt about the diagnosis, and of failing to notify the Health Department. On one occasion seven cases of smallpox in three neighbouring houses were allowed to continue undiagnosed and unnotified. It was only when two patients in the middle house became seriously ill that the medical attendant was alarmed and asked for the help and opinion of the Health Department.

**Smallpox at Nottingham—(*cont.*).**

It is easy to see that the work of Dr. Philip Boobyer, the Medical Officer of Health for Nottingham, and of Dr. Shaw, from the Ministry of Health, in preventing the spread of the disease, must have been most difficult on account of these atypical and missed cases by means of which the infection was kept alive. There are still some cases in the city, but they are now relatively few. There is no reason for supposing that smallpox will become epidemic: it has had its chance to be that and has not taken it. The disease might, however, become endemic, like chicken-pox, and be a cause of considerable financial loss to the town. As the disease is widespread, the complete vaccination of the people in the town would seem to be a desirable measure. Several thousand vaccinations have already been performed, and it is of interest to note that of the primary vaccinations with Government lymph 99.8 per cent. were successful, and of the secondary vaccinations no less than 98.3 succeeded.

### THE LOCAL COMMITTEE AND LEICESTER.

**(From a Correspondent.)**

AT a meeting of the Ashby-de-la-Zouch Urban Council, which was addressed on behalf of the Leicester Royal Infirmary's unit scheme to raise £100,000, Dr. Logan, medical officer to the local authorities, recently complained that the infirmaries in large towns were piling up work, congesting the hospitals, and absorbing all the local money. The Leicester Royal Infirmary, he said, was doing much work which the county cottage hospitals could do, thereby impoverishing the latter, and receiving many cases which had no right to go there.

When pressed to explain why these cases were so sent, Dr. Logan replied, "Never mind why: they do go there, and thus waste money and time in fares and trains."

In regard to the Leicester Infirmary scheme, Dr. Logan said that, in return for the support which the infirmary received from Ashby-de-la-Zouch, it should repay a proportion to the Ashby Cottage Hospital, indeed to all the cottage hospitals of the county, from which large sums would be received.

We note this recent criticism for several reasons. First, most large hospitals complain that too many cases are sent to them and do not wish to lengthen their existing waiting-lists; secondly, the cottage hospitals are entitled to a fair hearing in this matter. But the third reason is the chief:

That a medical man in the Public Health Service could make such a criticism proves, if more proof were needed, that there is a lack of co-ordination between our hospitals in the country areas, and that overlapping and competition are prominent. To these patent facts many hospital officers are still blind. The only practical remedy yet suggested is the formation of the new Local Committees, advocated by the Cave Committee, and now being formed under the Hospitals Commission.

Yet our last two issues gave abundant and distressing witness to the suspicion in which certain voluntary hospitals regard the Local Committees, though their main work is only to co-ordinate hospitals in their respective areas.

The moral therefore is, whatever be the rights and wrongs of Dr. Logan's criticisms concerning the present relation of the Leicester Royal Infirmary to the cottage hospitals of the county, that there is a case for sympathetic inquiry, and that the proper body to conduct it is the new Local Committee, formed, or in process, under the Voluntary Hospitals Commission. Before that body, the Royal Infirmary and the cottage hospitals could easily accommodate their policy and needs, if, as we cannot doubt, there is mutual desire for mutual satisfaction, in the public interest.

### MOVE FOR HOSPITAL ECONOMY.

**The Birmingham Plan.**

An important meeting has been held by the Hospitals Council in Birmingham, over which Sir David Brooks presided, to discuss how best to run the hospitals more economically.

The Finance Committee presented a report on co-operative purchase, and on the advantages obtainable by a comparison of the prices paid by hospitals for food, coal, drugs, and dressings. At present statistics showed a wide variation in the prices paid, hence the recommendation mentioned.

In regard to the Approved Societies, it is understood that in Birmingham some Approved Societies have received the sanction of the Ministry of Health to give grants to hospitals, in addition to "the maintenance of patients." The Council resolved to make further inquiries, but no public mention appears to have been made of the scheme on which the British Hospitals Association has been seeking the opinion of all hospitals affiliated to it, among which, of course, are the Birmingham hospitals.

The Birmingham Hospitals Council appears to share the fashionable dread of the Local Committees set up under the Cave Committee's Report, because it fears that some of its functions may be taken over by the new Local Committee.

### EDUCATIONAL CAMPAIGN AGAINST CANCER.

The American Society for the Control of Cancer was responsible for the National Cancer Week held throughout the United States and Canada from October 30 to November 5, 1921. This was the first attempt on the part of the Society to carry out a uniform campaign at one time throughout the country. The aim of this campaign was entirely educational, and it was desired to reach all sections of the country and as large a part of the population as possible with the message of cancer control. The three principal educational activities were (1) The giving of lectures by well-known physicians and surgeons, who volunteered for this service; (2) the distribution of literature at all meetings held during the week; (3) suitable publicity in the lay press, official health bulletins, and articles in professional journals. For the purpose of this campaign the American Society for the Control of Cancer organised campaign committees in practically all communities of 5,000 or more population.

